# Antimicrobial Susceptibility of *Escherichia coli* and *Salmonella* spp. Isolates From Healthy Pigs in Australia: Results of a Pilot National Survey

**DOI:** 10.3389/fmicb.2018.01207

**Published:** 2018-07-09

**Authors:** Amanda K. Kidsley, Sam Abraham, Jan M. Bell, Mark O'Dea, Tanya J. Laird, David Jordan, Pat Mitchell, Christopher A. McDevitt, Darren J. Trott

**Affiliations:** ^1^School of Animal and Veterinary Sciences, University of Adelaide, Roseworthy, SA, Australia; ^2^Australian Centre for Antimicrobial Resistance Ecology, University of Adelaide, Adelaide, SA, Australia; ^3^Antimicrobial Resistance and Infectious Diseases Laboratory, School of Veterinary and Life Sciences, Murdoch University, Perth, WA, Australia; ^4^New South Wales Department of Primary Industries, Wollongbar, NSW, Australia; ^5^Australian Pork Limited, Canberra, ACT, Australia; ^6^Research Centre for Infectious Diseases, School of Biological Sciences, University of Adelaide, Adelaide, SA, Australia

**Keywords:** antimicrobial resistance, *Escherichia coli*, food-producing animals, fluoroquinolones, critically important antimicrobials

## Abstract

This study investigated the frequency of antimicrobial non-susceptibility (defined as the frequency of isolates with minimum inhibitory concentrations above the CLSI susceptible clinical breakpoint) among *E. coli* and *Salmonella* spp. isolated from healthy Australian finisher pigs. *E. coli* (*n* = 201) and *Salmonella* spp. (*n* = 69) were isolated from cecal contents of slaughter-age pigs, originating from 19 farms distributed throughout Australia during July-December 2015. Isolates underwent minimum inhibitory concentration (MIC) susceptibility testing to 11 antimicrobials. The highest frequencies of non-susceptibility among respective isolates of *E. coli* and *Salmonella* spp. were to ampicillin (60.2 and 20.3%), tetracycline (68.2 and 26.1%), chloramphenicol (47.8 and 7.3%), and trimethoprim/sulfamethoxazole (33.8 and 11.6%). Four *E. coli* isolates had MICs above the wild-type epidemiological cut-off value for ciprofloxacin, with two isolates from the same farm classified as clinically resistant (MICs of > 4 μg/ml), a noteworthy finding given that fluoroquinolones (FQs) are not legally available for use in Australian food-producing animals. Three of these four *E. coli* isolates belonged to the sequence type (ST) 10, which has been isolated from both humans and production animals, whilst one isolate belonged to a new ST (7573) and possessed *qnrS1*. This study shows that non-susceptibility to first line antimicrobials is common among *E. coli* and *Salmonella* spp. isolates from healthy slaughter age pigs in Australia. However, very low levels of non-susceptibility to critically important antimicrobials (CIAs), namely third generation cephalosporins and fluoroquinolones were observed. Nevertheless, the isolation of two ciprofloxacin-resistant *E. coli* isolates from Australian pigs demonstrates that even in the absence of local antimicrobial selection pressure, fluoroquinolone-resistant *E. coli* clonal lineages may enter livestock production facilities despite strict biosecurity.

## Introduction

*Escherichia coli* and *Salmonella* spp. while common commensals in many animals, are also known to be the causative agents of a number of production limiting diseases in pigs (Quinn et al., 2011). *E. coli* can cause pre-weaning scours and septicemia in piglets, post-weaning diarrhea and edema disease in weaners, and mastitis and cystitis in sows (Zimmerman et al., 2012). Enterotoxigenic *E. coli* (ETEC), the main agent associated with post-weaning enteric colibacillosis, is among the most significant bacterial pathogens in Australian pig production and is commonly resistant to multiple antimicrobial agents (Smith et al., 2016).

*Salmonella enterica*, subspecies *enterica* one of the primary subspecies of *Salmonella* associated with foodborne disease and a well-known zoonotic pathogen, is commonly carried by pigs and other food-producing animals (Abraham et al., 2014b). High rates of multidrug resistance have been found in *Salmonella* iolates from food-producing animals in several countries. For example, 54.5–55.6% of *Salmonella* spp. isolates from bovine carcasses in Croatia and Spain (European Food Safety Authority (EFSA), and European Centre for Disease Prevention and Control (ECDC), 2017); 66% of *Salmonella* spp. isolates from poultry and swine in Thailand (Van et al., 2012); and 41% of turkey, 8.3% of chicken and 17% of cattle *Salmonella* spp. isolates in the United States (Centers for Disease Control and Prevention (CDC), U.S. Department of Agriculture(USDA), and Food and Drug Administration (FDA), 2014) are multidrug-resistant (MDR). A recent Australian study found a high proportion (66.1%) of clinical *Salmonella* spp. isolates from food-producing animals, the majority obtained from bovine sources, were susceptible to all antimicrobials tested, including to critically important antimicrobials (CIAs), namely extended-spectrum cephalosporins (ESCs) and fluoroquinolones (FQs) (Abraham et al., 2014a). This low rate of resistance among bovine origin *Salmonella* spp. isolates was also confirmed in a study of *Salmonella* carriage in healthy cattle at slaughter (Barlow et al., 2015). However, ESC-resistant *Salmonella* spp. strains have recently been isolated from Australian dairy cattle in Gippsland, Victoria (Sparham et al., 2017). Although Australian pigs have previously been considered to have low rates of *Salmonella* spp. infection, since 2011 increasing numbers of clinical cases have been reported (Hamilton et al., 2015). Despite this, there have been no published studies on the estimated prevalence of antimicrobial resistance (AMR) in *Salmonella* spp. isolated from healthy Australian pigs at slaughter.

Antimicrobial agents are vital for the treatment and control of many bacterial diseases in pig production (Smith et al., 2016), but widespread use is often associated with the selection of AMR (Smith et al., 2016). MDR pathogens in humans, companion, and food-producing animals are a potential threat to animal health through the loss of antibiotic effectiveness to treat diseases and also to human health via direct cross-infection or foodborne transmission of organisms such as *Salmonella* spp. or indirectly through the transfer of mobile genetic elements, such as plasmids, between bacteria (Jordan et al., 2009; Mukerji et al., 2017). The reported use of antimicrobials and associated resistance in food-producing animals differs throughout the world. European AMR surveillance data show large differences between countries in both their antimicrobial use and frequency of AMR in key indicator bacteria (Österberg et al., 2016). For example, in respective studies undertaken in Italy and Poland, 12% (*n* = 125) (Österberg et al., 2016) and 11.1% (*n* = 190) (Wasyl et al., 2013) of *E. coli* isolated from the feces of healthy pigs close to slaughter weight were resistant or non-wild type to ciprofloxacin respectively. In contrast, similar studies undertaken in Canada during 2013 and Sweden in 2015, reported frequencies of resistance and non-wild type to ciprofloxacin of 2.4% (*n* = 171) and 2.5% (*n* = 200), respectively (Government of Canada, 2015; Swedish Veterinary Antimicrobial Resistance Monitoring (SVARM), 2015). In France, the frequency of ciprofloxacin resistance in *E. coli* isolated from porcine colonic contents at slaughter was 4.3% (*n* = 94). Interestingly, no ciprofloxacin resistance was reported from isolates collected during a contemporaneous study in Denmark (*n* = 52) (Österberg et al., 2016).

Australia was recently ranked the 5th lowest user of antimicrobials in livestock (mg/kg) in the world (O'Neill, 2015), which may in part be due to its heavy reliance on extensive grazing systems, but could also be related to other factors. Antimicrobials approved in Australia for the treatment of infections in pigs cover a broad range of classes and include sulphonamide-trimethoprim combinations, tetracyclines, β-lactams, and aminoglycosides (neomycin, apramycin, and spectinomycin) (Smith et al., 2016). Other antimicrobials, such as the ESC ceftiofur and the phenicol florfenicol, can be used by Australian veterinarians “off label” for individual cases of porcine colibacillosis as they are only approved for use for respiratory infections in cattle (ceftiofur), and both cattle and pigs (florfenicol) (Smith et al., 2016). In contrast to several other countries, the use of CIAs in Australian livestock is highly regulated (Smith et al., 2016; Mukerji et al., 2017). Australia is the only country to implement legal measures that exclude the use of FQs and gentamicin in food-producing animals (Abraham et al., 2014a). Further, no product containing colistin has been registered for use in Australian livestock for over 25 years (Australian Pesticides and Veterinary Medicine Authority (APVMA), 2017). In addition, by international comparison, the label constraints on the use of ESCs in Australian livestock are strict, while the ESC cefquinome is not registered for use. However, in a 2006 study, off label use of ceftiofur was reported to have occurred on 25% of Australian piggeries (Jordan et al., 2009).

Minimum inhibitory concentration (MIC) testing of commensal bacteria from healthy animals is commonly used to evaluate the occurrence of AMR in animal populations and farms and is the basis for mandatory monitoring of food production animals in the European Union (EU) (Österberg et al., 2016). Importantly, commensal bacteria such as *E. coli* can be reservoirs of plasmid-associated resistance genes of public health significance (Trott, 2013). While proof of concept national AMR surveys in the various livestock sectors have commenced (Shaban et al., 2014), a number of opportunistic surveys conducted in recent years have confirmed a low public health risk in the Australian food animal sector in relation to resistance to CIAs, such as FQs and ESCs (Abraham et al., 2012, 2014a, 2015; Barlow et al., 2015). However, given the critical differences between the antimicrobial use in the Australian pig industry and elsewhere, and the lack of contemporary information on the occurrence of resistance, the aim of this pilot study was to investigate the occurrence of AMR among commensal *E. coli* and *Salmonella* spp. isolated from cecal contents of Australian finisher pigs at slaughter. The frequency of isolates with MICs classified as non-susceptible based on Clinical Laboratory Standards Institute (CLSI) and National Antimicrobial Resistance Monitoring System (NARMS) clinical breakpoints was determined. In addition, isolates with MICs above the wild-type epidemiological cut off values (ECOFFs) for CIAs were further characterized by whole genome sequencing analysis.

## Materials and methods

### Sample collection, isolation, and identification

All cecal specimens were obtained using a systematic-random sampling method from healthy pigs at slaughter originating from 19 farms distributed throughout Australia between July and December 2015. Abattoirs were identified based on their eligibility criteria (e.g., export abattoirs processing finishing pigs where a Department of Agriculture on-plant veterinarian was present) and then randomly selected. The number of animals sampled from each abattoir was proportional to the output of that establishment, and calculated in advance. A systematic-random method of sampling was used with samples collected at regular intervals along the chain throughout the day. The interval between collections of individual samples for each plant was calculated (approx.) as a function of chain speed, daily throughput and shift length. A total of 201 pigs were sampled with one sample per pig obtained after slaughter and scalding when the gastrointestinal tract was removed. Samples were stored at 2–4°C before being packed and shipped with samples arriving at the primary laboratory within 24 h of collection.

A 10 g sample of fecal material was suspended in 7 ml of 0.1% sterile buffered peptone water (BPW) and thoroughly mixed, before 1 ml of the fecal mixture was extracted and centrifuged. The homogenate was plated on to MacConkey agar (Oxoid, Thermofisher Scientific) and incubated at 37°C for 18–24 h. Several lactose positive presumptive *E. coli* colonies were sub-cultured onto sheep blood agar (SBA) (Oxoid, Thermofisher Scientific) and incubated at 37°C for 24 h. One colony identified as *E. coli* using standard biochemical tests (Markey et al., 2013) was used for further analysis. For *Salmonella* isolation, the remaining fecal sample in BPW was incubated at 37°C for 18–24 h. Following incubation 10 ml of Rappaport-Vassiliadis broth (Micromedia, Edwards) was inoculated with 0.1 ml of the incubated buffered peptone water and incubated at 42°C for 18 h. An aliquot was then streak plated onto *Salmonella* Brilliance agar (Oxoid, Thermofisher Scientific) and XLD agar (Micromedia, Edwards) to select for single colonies and incubated at 37°C for 24 h. Well isolated single colonies were sub-cultured onto SBA and incubated at 37°C for 24 h. These presumptive *Salmonella* spp. isolates were then confirmed biochemically (Markey et al., 2013), with one isolate per sample selected for further analysis. The identity of each bacterial isolate to species (*E. coli* and *Salmonella* spp.) level was confirmed using mass-spectrometry (MALDI-TOF) prior to antimicrobial susceptibility testing (AST).

### Antimicrobial susceptibility testing

AST was performed by micro-broth dilution using commercially prepared dryform panels (Sensititre CMV3AGNF, NARMS; Trek Diagnostic Systems, Thermofisher Scientific). Inoculation and incubation was carried out as per the manufacturer's guidelines, with quality control strains *E. coli* ATCC 35218, *E. coli* ATCC 25922, *Enterococcus fecalis* ATCC 29212, *Staphylococcus aureus* ATCC 29213, and *Pseudomonas aeruginosa* ATCC 27853 used throughout the study. The antimicrobials tested were ampicillin, amoxicillin/clavulanic acid, cefoxitin, ceftiofur, ceftriaxone, chloramphenicol, ciprofloxacin, gentamicin, streptomycin, tetracycline and trimethoprim/sulfamethoxazole and were selected based on consultation with industry and their widespread use in international antimicrobial resistance surveillance programmes (Shaban et al., 2014). MICs were interpreted using CLSI VET01S (Clinical Laboratory Standard Institute, 2015) guidelines or NARMS guidelines (Centers for Disease Control and Prevention (CDC), U.S. Department of Agriculture(USDA), and Food and Drug Administration (FDA), 2014) where no interpretative criteria were available (Table 1). In addition, CLSI M100S (Clinical Laboratory Standards Institute, 2016) breakpoints were used where animal species specific breakpoints were not available. Isolates with MICs above the susceptible breakpoint (i.e., in the intermediate or resistant category) were classified as non-susceptible (Clinical Laboratory Standards Institute, 2011). Resistance profiles were generated, with isolates classified as MDR if they showed non-susceptibility to one antimicrobial agent in three or more antimicrobial classes (Magiorakos et al., 2012). The European Committee on Antimicrobial Susceptibility Testing (EUCAST) ECOFFs were used to select isolates with an MIC value above the wild-type for ESCs and FQs.

**Table 1:** Breakpoints used for AST testing of *E. coli* and *Salmonella* spp. isolates.

**Antimicrobial class**	**Antimicrobial agent**	**Range (μg/ml)**	**ECOFF^a^**		**CLSI^b^ or NARMS^c^**		
			***E. coli***	***Salmonella* spp**.	**S**	**I**	**R**
Aminoglycosides	Gentamicin	0.25–16	2	2	≤ 4	8	≥16
	Streptomycin	2–64	16	16	≤ 32	–	> 32
β-lactam / β-lactam inhibitor combinations	Amoxicillin-clavulanate	1–32	–^d^	–	≤ 8	16	≥32
Cephems	Cefoxitin	0.5–32	8	8	≤ 8	16	≥32
	Ceftiofur	0.12–8	1	2	≤ 2 ^e^	4	≥8
	Ceftriaxone	0.25–64	0.12	–	≤ 1	2	≥4
Fluoroquinolones	Ciprofloxacin (*E. coli*)	0.015–4	0.06	0.06	≤ 1	2	≥4
	Ciprofloxacin (*Salmonella* spp.)	0.015–4	0.06	0.06	≤ 0.06	0.12–0.5	≥1
Folate pathway inhibitors	Trimethoprim-sulfamethoxazole	0.12–4	1	1	≤ 2	–	≥4
Penicillins	Ampicillin	1–32	8	8	≤ 8	16	≥32
Phenicols	Chloramphenicol	2–32	16	16	≤ 8	16	≥32
Tetracyclines	Tetracycline	4–32	8	8	≤ 4	8	≥16

a *EUCAST epidemiological cut-off values (μg/ml)*.

b *CLSI VET01S, or M100S breakpoints (μg/ml), S = sensitive; I = intermediate; R = resistant*.

c *NARMS breakpoints (μg/ml) (in blue)*.

d *not defined*.

e *E. coli only*.

### Whole genome sequencing

Whole genome sequencing was performed on eight isolates that had an ECOFF value above the wild-type for either ESCs or FQs, using Illumina MiSeq as described by Worthing et al. (2017). Briefly, samples underwent library preparation using the Nextera XT DNA library preparation kit according to the manufacturer's instructions, and sequencing was performed on a MiSeq V3 2x300 flow cell. The Nullarbor pipeline v1.01 (https://github.com/tseemann/nullarbor) was used to assemble the eight Illumina sequenced strains. The resulting FASTA files were analyzed using the ResFinder, VirulenceFinder and PlasmidFinder functions of the Centre for Genomic Epidemiology database (http://www.genomicepidemiology.org/).

## Results

### Culture results

*E. coli* was isolated from all porcine cecal samples collected (*n* = 201). In contrast, *Salmonella* spp. were only recovered from cecal samples from 14 of the 19 (73.7%) farms sampled (*n* = 69 isolates).

### Phenotypic antimicrobial resistance characterization

**Figure 1:**
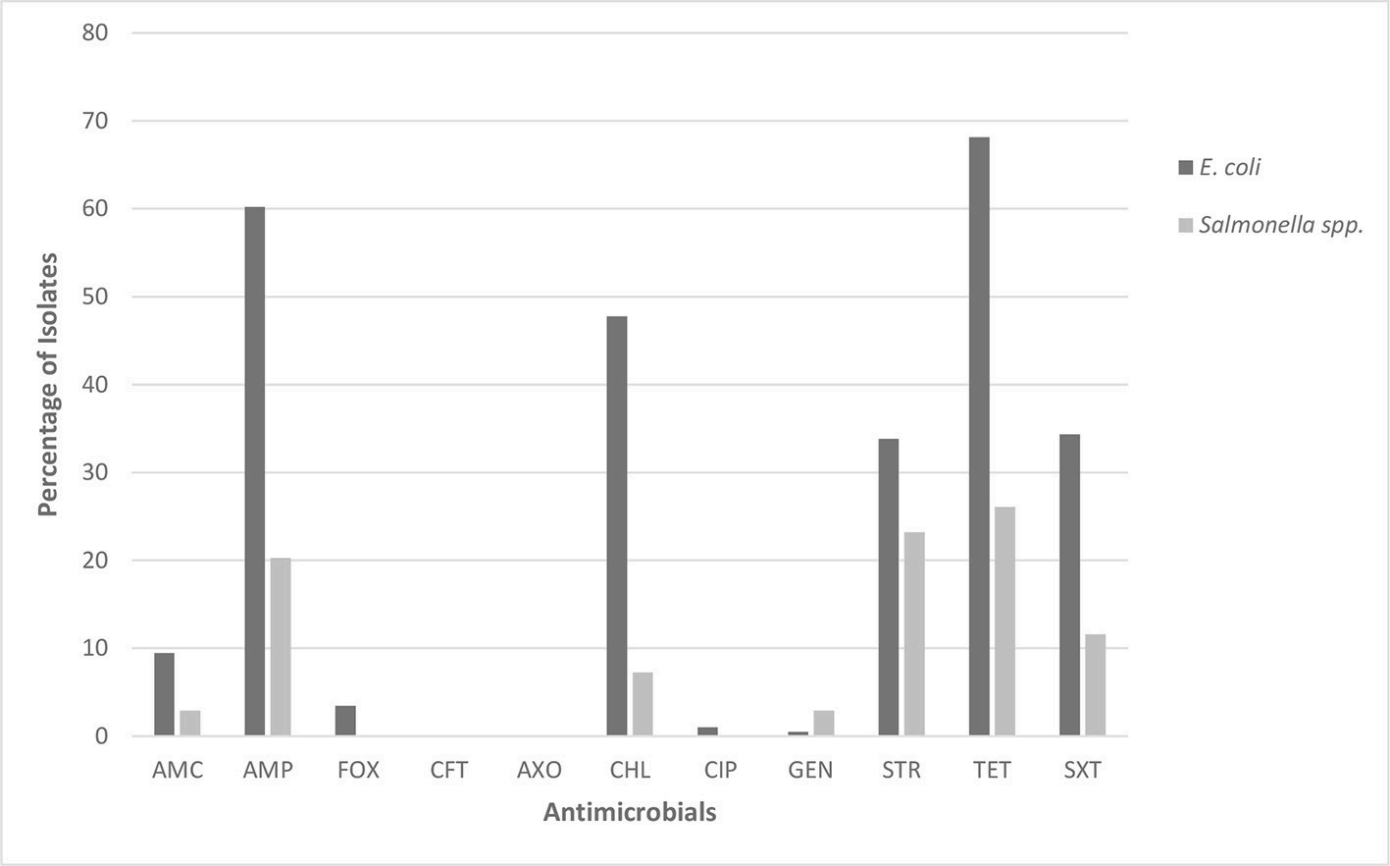
Percentage of *E. coli* (*n* = 201) and *Salmonella* spp. (*n* = 69) isolates showing non-susceptibility to 11 selected antimicrobials. AMC, amoxicillin/clavulanic acid; AMP, ampicillin; FOX, cefoxitin; CFT, ceftiofur; AXO, ceftriaxone; CHL, chloramphenicol; CIP, ciprofloxacin; GEN, gentamicin; STR, streptomycin; TET, tetracycline; SXT, trimethoprim/sulfamethoxazole.

The 201 *E. coli* isolates showed the highest levels of non-susceptibility to ampicillin (60.2%), tetracycline (68.2%), chloramphenicol (47.8%) and trimethoprim/sulfamethoxazole (34.3%) (Figure 1). By contrast, although the 69 *Salmonella* spp. isolates also showed the highest levels of non-susceptibility to ampicillin and tetracycline, these had a lower frequency of occurrence (20.3 and 26.1%, respectively). Furthermore, *Salmonella* spp. isolates had lower levels of non-susceptibility to trimethoprim/sulfamethoxazole (11.6%) and chloramphenicol (7.3%). Fifty-one percent of *E. coli* and 21.7% of *Salmonella* spp. isolates were classified as MDR.

Low levels of non-susceptibility were observed among isolates of both species to amoxicillin/clavulanate (*E. coli* 9.5%; *Salmonella* spp. 2.9%) and gentamicin (*E. coli* 0.5%; *Salmonella* spp. 2.9%). Overall, low levels of non-susceptibility were detected to antimicrobials classified as critically important to human health (ESCs and FQs). Ceftiofur non-susceptibility was not observed for either *E. coli* or *Salmonella* spp. However, two *Salmonella* spp. isolates (2.9%) were found to have MIC values above the wild-type ECOFF (Table 2). Four *E. coli* isolates (2.0%) had ciprofloxacin MICs above 0.25 μg/ml, which is also above the wild-type ECOFF (MIC > 0.06 μg/ml). However, only two of these isolates, both obtained from the same farm (farm Q), had MICs above the CLSI resistant clinical breakpoint (MICs of > 4 μg/ml), despite no reported usage of FQs on this farm according to the Australian Pork Industry Quality Assurance Program (APIQ) audits. In addition, two *E. coli* isolates (1.0%) had cefoxitin MICs above the wild-type ECOFF and were classified as non-susceptible on the basis of CLSI clinical breakpoints. All isolates from farm Q (*n* = 12) were also classified as MDR. One *E. coli* isolate (MIC above the cefoxitin wild-type ECOFF) and one *Salmonella* spp. isolate (MIC above the ceftiofur wild-type ECOFF) were isolated from the same farm (farm D). All other isolates showing non-susceptibility to ESCs and/or FQs were obtained from different farms [farms H, K, R, and X (*n* = 1 for all farms)]. One of the *Salmonella* spp. isolates with a MIC value above the wild-type ECOFF for ceftiofur showed susceptibility to all other antimicrobials tested (farm H). The other seven isolates of interest were classified as MDR.

**Table 2:**
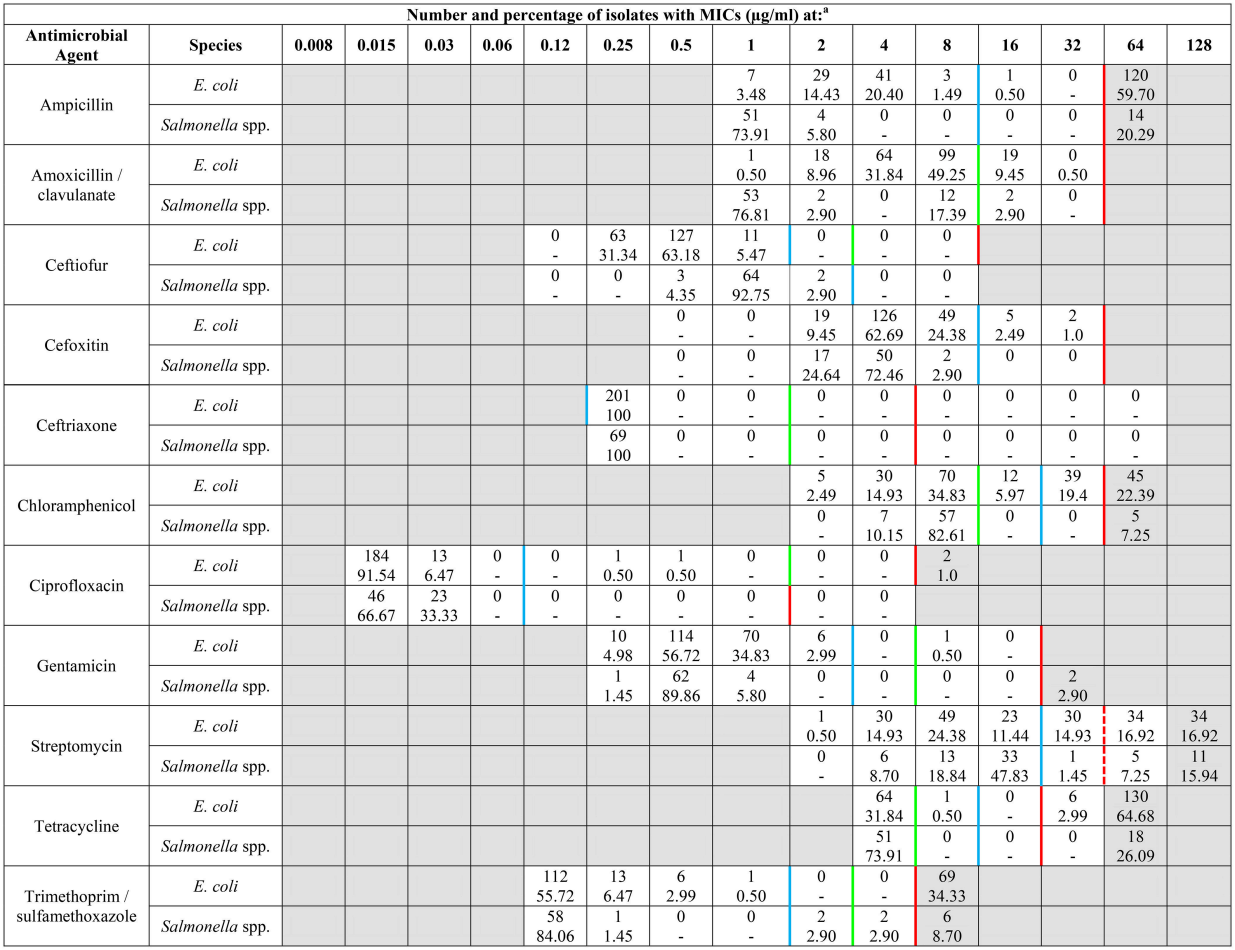
MIC distribution frequency of *E. coli* (*n* = 201) and *Salmonella* spp. (*n* = 69) isolates.

*^a^Unshaded areas indicate MIC range for each agent available on the Sensititre CMV3AGNF card. MICs > than highest concentration available are indicated in the shaded region. Vertical blue lines indicate EUCAST ECOFF values; CLSI susceptible (green) and resistant (red) breakpoints; and NARMS breakpoints (red dashes)*.

**Figure 2:**
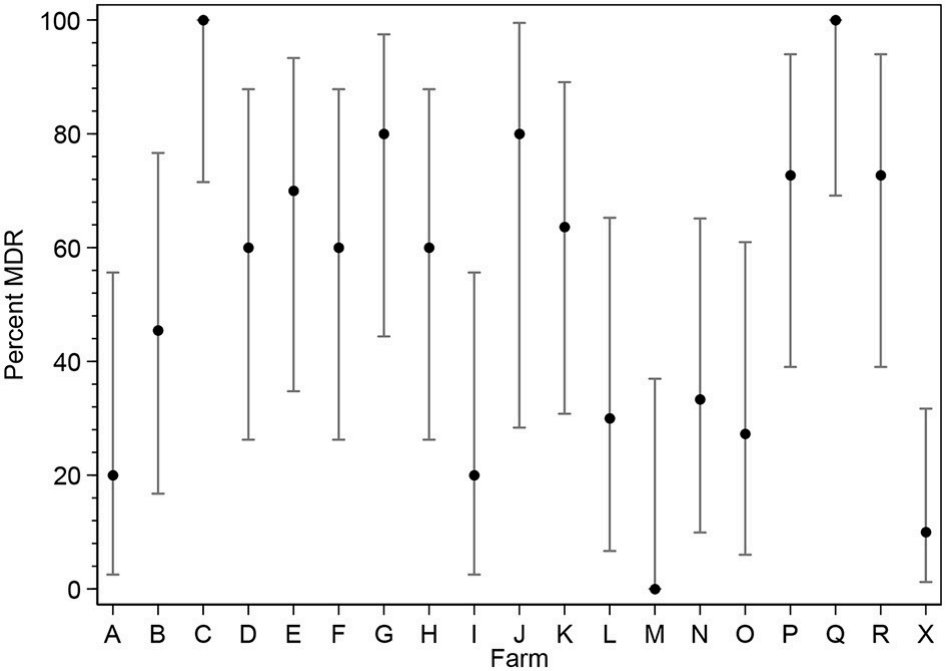
Point estimates and 95% confidence interval for the percent of commensal *E. coli* colonies expressing MDR phenotype within each of 19 Australian pig herds.

The percentage of MDR *E. coli* isolates was further broken down and analyzed on a per farm basis (Figure 2). Although the sample size per farm is limited, there is sufficient evidence to suggest there is a large variation between farms in the underlying proportion of *E. coli* that are MDR. The *Salmonella* spp. were unable to be analyzed by farm due to the lower number of isolates obtained. As shown in Table 3 the most common MDR profile was non-susceptibility to β-lactam/β-lactam inhibitor combinations, phenicols, aminoglycosides, tetracyclines and folate pathway inhibitors; followed by non-susceptibility to β-lactam/β-lactam inhibitors, phenicols and tetracyclines.

**Table 3:** Resistant and MDR profiles with the highest frequency in *E. coli* (*n* = 201) and *Salmonella* spp. (*n* = 69).

**Number of antimicrobial classes**	**Total no. of isolates (%)**		**Resistance pattern (no. of isolates)**	
	***E. coli***	***Salmonella*** **spp**.	***E. coli***	***Salmonella*** **spp**.
All susceptible	26 (12.94)	46 (66.67)	26	45
1	33 (16.42)	6 (8.70)	TET (20)	SXT (3) TET (3)
2	40 (19.90)	2 (2.90)	BLA-TET (18)	AMG-BLA (1) CHL-SXT (1)
3	36 (17.91)	11 (15.94)	BLA-CHL-TET (13)	AMG-BLA-TET (9)
4	40 (19.90)	1 (1.45)	BLA-CHL-SXT-TET (11) AMG-CHL-SXT-TET (11)	AMG-BLA-SXT-TET (1)
5	20 (9.95)	2 (2.90)	AMG-BLA-CHL-SXT-TET (14)	AMG-BLA-BLI-SXT-TET (1) AMG-BLA-CHL-SXT-TET(1)
6	3 (1.49)	1 (1.45)	AMG-BLA-BLI-CHL-SXT-TET (2)	AMG-BLA-BLI-CHL-SXT-TET (1)
7	3 (1.49)	0 (0)	AMG-BLA-BLI-CHL-2GC-SXT-TET (2)	-
Non-MDR	99 (49.25)	54 (78.26)	99	54
MDR	102 (50.75)	15 (21.74)	102	15

*Antimicrobial categories included: aminoglycosides, AMG (gentamicin, streptomycin); penicillin, BLA (ampicillin); β-lactam inhibitors, BLI (amoxicillin-clavulanate); phenicols, CHL (chloramphenicol); 2nd generation cephalosporins, 2GC (cefoxitin); folate pathway inhibitors, SXT (trimethoprim/sulfamethoxazole); and tetracycline, TET*.

### Molecular characterization

Eight isolates were selected for whole genome sequencing based on having MICs at or above the ECOFF for CIAs. These comprised the two *E. coli* isolates that were clinically resistant to FQs from farm Q (ciprofloxacin MICs > 4 μg/ml), two *E. coli* isolates with ciprofloxacin MICs of 0.25 μg/ml and 0.5 μg/ml, two *E. coli* isolates with cefoxitin MICs of 32 μg/ml, and two *Salmonella* isolates with ceftiofur MICs of 2 μg/ml (Table 4).

**Table 4:** Comparison of AMR genes, virulence genes, plasmids and multilocus sequence type (MLST) in eight isolates with reduced susceptibility to ESCs and/or FQs.

**Isolate**	**Species**	**MIC values**	**MLST**	**Resistance genotype**	**Plasmids**	**Virulence genes**	**QRDR mutations**	
							**QRDR**	**Amino acid substitution**
1	*E. coli*	FOX 32	10	*bla_*TEM*−1*B*_, tet(B)*	*IncR, IncY*	*gad*	Not determined	
2	*E. coli*	CIP 0.25	New (7573)	*aadA2, aadA1, bla_*TEM*−1*B*_, qnrS1, floR, cmlA1, sul3, sul2, tet(A), tet(M), dfrA12*	*IncFIB(AP001918), Col8282, p0111, IncFIA(HI1), IncHI1B(R27), IncHI1A*	*mchC, ipfA, mchB, iss, mcmA, mchF, astA*	Not Detected	
3	*E. coli*	CIP ≥4	10	*aadA1, aph(3')-la, bla_*TEM*−1*B*_, sul3, tet(A), tet(M), dfrA1*	*IncFll, IncX1, IncR*	*iss, gad*	DNA gyrase A subunit (GyrA)	Ser(83)—Leu
								Asp (87)—Asn
								Glu (678)—Asp
								Ser (828)—Ala
							DNA gyrase B subunit (GyrB)	Asp(185)—Glu
							Topoisomerase IV A subunit (ParC)	Ser(80)—Ile
								Glu(475)—Asp
							Topoisomerase IV B subunit (ParE)	Ile(136)—Val
4	*E. coli*	CIP ≥4	10	*aadA1, aph(3′)-la, bla_*TEM*−1*B*_, sul3, tet(A), tet(M), dfrA1*	*IncX1, IncFll, IncR*	*iss, gad*	DNA gyrase A subunit (GyrA)	Ser(83)—Leu
								Asp (87)—Asn
								Glu (678)—Asp
								Ser (828)—Ala
							DNA gyrase B subunit (GyrB)	Asp(185)—Glu
							Topoisomerase IV A subunit (ParC)	Ser(80)—Ile
								Glu(475)—Asp
							Topoisomerase IV B subunit (ParE)	Ile(136)—Val
5	*E. coli*	CIP 0.5	10	*aadA2, aadA1, bla_*TEM*−1*A*_, mef(B), cmlA1, sul3, tet(A), dfrA12*	*p0111, IncX4, IncFIA(HI1), IncFIB(K)*	*celb, gad*	DNA gyrase A subunit (GyrA)	Ser(83)—Leu
								Glu (678)—Asp
								Ser (828)—Ala
							DNA gyrase B subunit (GyrB)	Asp(185)—Glu
							Topoisomerase IV A subunit (ParC)	Glu(475)—Asp
							Topoisomerase IV B subunit (ParE)	Ile(136)—Val
6	*E. coli*	FOX 32	4417	*aadA2, strA, strB, sul3, sul1, tet(A), dfrA12*	*Col(MG828), IncY*	*gad, celb,ipfA*	Not determined	
7	*Salmonella spp*.	CEF 2	469	*aadA2, strA, aph(3′)-lc, strB, bla_*TEM*−1*B*_, catA1, floR, sul1, sul2, tet(D), dfrA12*	*Col(MG828), IncA/C2*	*celb*	Not determined	
8	*Salmonella spp*.	CEF 2	515	*dfrA5*	*IncFII(29), IncFIB(AP001918)*	-	Not determined	

*Virulence gene functions- gad: glutamate decarboxylase enzyme associated with acid tolerance; ipfA: adhesion that encodes long polar fimbriae that plays integral role in attachment of EPEC to the gut wall (Blum and Leitner, 2013) and can cause diarrhea in humans; iss: encodes increased serum survival protein which increases survival in animal serum (Miajlovic and Smith, 2014) and is recognized for its role in ExPEC virulence; mcmA: a microcin precursor gene encoding microcin M. Microcins are antimicrobial peptides secreted by members of the Enterobacteriaceae family and are involved in microbial competition within the intestinal tract (Vassiliadis et al., 2010); astA: encodes for heat stable enterotoxin EAST1 that is found mostly in enterohemorrhagic and enteroaggegative E. coli; celb encodes for the induction of cell lysis and the subsequent release of the endonuclease colicin E2 in E. coli (Mader et al., 2015); mchC, mchB and mchF are all involved in the production of microcin peptide antibiotics called in enterobacterial strains. mchB encodes for the synthesis of the microcin H47 (MccH47) peptide precursor. The product of mchC and other mch genes carry out the process of maturation of MccH47by adding an enterobactin derivative which is employed for MccH47 synthesis. After synthesis MccH47 is secreted through a type I apparatus that is formed by products of mchF and another mch gene (Poey et al., 2006)*.

Four of the six *E. coli* isolates belonged to *E. coli* sequence type (ST) 10, which belongs to phylogenetic group A and is commonly isolated from a range of animal species as well as humans. All *E. coli* ST10 isolates were classified as MDR and possessed at least one β-lactamase gene, but no ESC resistance-associated genes were identified in these isolates. The three ST10 isolates with the highest ciprofloxacin MICs had similar amino acid substitutions in the quinolone resistance-determining regions (QRDRs) of DNA gyrase A subunit (GyrA) and topoisomerase IV A subunit (ParC). The main substitutions of note were S83L and D87N in GyrA and S80I in ParC. Although other point mutations were identified in GyrB (A185G) and ParE (I136V), these are not typically associated with quinolone resistance. Whilst it is possible that additional mechanisms of FQ resistance, such as overexpression of efflux pumps may be present in the two ST10 isolates with ciprofloxacin MICs > 4.0 μg/ml, their genomes were not interrogated further.

The remaining *E. coli* isolates belonged to a new sequence type, designated ST7573, and ST4417 (Table 4). The isolate belonging to ST7573 was the only isolate that contained a plasmid-mediated quinolone resistance (PMQR) gene (*qnrS1*). However, this isolate was still classified as ciprofloxacin-susceptible according to CLSI guidelines, and did not possess any identifiable chromosomally-encoded FQ resistance mechanisms. Further, this isolate was MDR and contained the extraintestinal pathogenic *E. coli* (ExPEC) virulence factor gene *iss*, which encodes for increased serum survival, in addition to a range of microcin-associated genes, the EAST-1 toxin (*astA*) and long polar flagella genes (*ipfA*) (Table 4). However, this particular combination of *E. coli* virulence genes does not classify the ST7573 isolate as belonging to any particular *E. coli* pathotype.

The *iss* virulence associated gene was also identified in two *E. coli* isolates belonging to ST10 together with *gad*, a glutamate decarboxylase gene involved in acid tolerance. Apart from the cellobiose utilization gene *celB* being identified in one ST10 isolate and the single ST4417 isolate, no other ExPEC-associated virulence genes were identified in any of the isolates subjected to whole genome sequence analysis.

The two *Salmonella* spp. isolates belonged to ST469 (serotype Rissen), a commonly distributed serotype previously associated with pig production, and ST515 (serotype Johannesburg).

Isolate sequences were deposited in Enterobase with the accession numbers: traces-0GpondC, traces-0GQRdxI, traces-0YZcnKW, traces-0ILjJIq, traces-0bhIgfw, and traces-0fwHIrT (*E. coli* isolates 1, 2, 3, 4, 5, and 6 respectively) and traces-0OMUTNy and traces-0LIahFC (*Salmonella* spp. isolates 1 and 2).

## Discussion

The main aims of this study were to investigate the occurrence of AMR among *E. coli* and *Salmonella* spp. isolated from healthy Australian finisher pigs at slaughter and further characterize any isolates found to be non-susceptible to CIAs (ESCs and FQs) using whole genome sequencing. The major findings from this study are: (1) Low levels of non-susceptibility to CIAs were detected among both *E. coli* and *Salmonella* spp. isolates; (2) Of the eight isolates with MICs above the wild-type for either ciprofloxacin, cefoxitin or ceftiofur, four *E. coli* isolates belonged to ST10 including two isolates that were clinically resistant to ciprofloxacin and one *Salmonella* spp. isolate belonged to the internationally distributed ST469 associated with serotype Rissen; and (3) High frequencies of non-susceptibility were observed to antimicrobial classes with a lower importance rating (Australian Strategic and Technical Advisory Group on AMR (ASTAG), 2015; Australian Veterinary Association, 2015) that are registered for use in pigs in Australia (i.e., tetracyclines, aminopenicillins and sulphonamide/trimethoprim combinations).

Ceftiofur resistance was previously reported in porcine commensal *E. coli* isolated from 1.8% of pooled fecal samples from finisher pigs at Australian piggeries. However, none of the isolates possessed plasmid-mediated AmpC or extended-spectrum β-lactamases (ESBLs) (Smith et al., 2016). The first detection of ESC resistance associated with ESBLs in *E. coli* from Australian food-producing animals was reported in clinical isolates in 2015. A national survey of clinical isolates from diseased pigs obtained from veterinary diagnostic laboratories identified three porcine *E. coli* isolates (2.6%) as resistant to ceftiofur, with one isolate, identified as an ST774 strain, also exhibiting resistance to ciprofloxacin (Abraham et al., 2015). The frequency of ciprofloxacin non-susceptibility observed in the present study was also low (1%) with two isolates from farm Q both exhibiting ciprofloxacin MICs above the resistant clinical breakpoint (indicating that FQ-R ST10 was the dominant *E. coli* present in the gut of slaughter age pigs on this farm). This is a significant finding as it indicates the presence of resistance to FQs in commensal *E. coli* from Australian pigs despite the absence of direct on farm selection pressure. This correlates to data from a recent Danish Integrated Antimicrobial Resistance Monitoring and Research Programme (DANMAP) report where 1% of *E. coli* isolated from cecal samples randomly collected from healthy pigs at slaughter were identified as being ciprofloxacin non-wild-type (Høg et al., 2015). FQ resistance has previously been strongly correlated with the quantity of antimicrobials used in the treatment of pigs (Barton, 2014), so given the legal constraints on FQ use in Australian pigs it is possible that these isolates did not develop FQ resistance on farm and were likely introduced from an extraneous source. One hypothesis is that they may have been introduced via human carriers or wild birds, as suggested by Abraham and co-workers (Abraham et al., 2015), but other potential sources of transmission could also be considered such as feed, water, rodents and insects. This highlights the need for emphasis of biosecurity measures and their widespread application and extension to non-animal sources of AMR transmission, such as in-contact humans.

Resistance to ceftiofur in commensal bacteria (*Salmonella* and *E. coli*) isolated from pigs was first reported in 2002 in south-east Asia (Hanson et al., 2002). Other reports soon followed (Barton, 2014). A recent Australian regional survey of fecal samples from 22 Australian commercial pig farms found 5.2% of *E. coli* isolates were resistant to ceftiofur (van Breda et al., 2018). The emergence of some level of non-susceptibility to ceftiofur in Australian herds is not unexpected, given previous reporting of “off-label” use in individual pigs, which is assumed to be for the treatment of ETEC infection (Jordan et al., 2009), raising concerns of the potential for cross-transfer of ceftiofur non-susceptibility to humans via the food chain. However, the results of the current Australia-wide survey suggest that ceftiofur-resistant commensal *E. coli* are not dominant coliforms in the gut of slaughter age pigs that are likely to be isolated during AMR surveillance programs. Nevertheless, the emergence and recent detection of ceftiofur-resistant *E. coli* containing AmpC and ESBLs (Abraham et al., 2015; van Breda et al., 2018) confirms that off-label use of ESCs should be more critically evaluated by the Australian industry, as has recently occurred in Denmark (Agersø and Aarestrup, 2013). The Danish Agriculture and Food Council recommended a voluntary ban on the use of ESCs in pigs following increased detection of ESBL resistance genes in swine production facilities (Agersø and Aarestrup, 2013). It is noted that FQs are registered for use in food-producing animals in many European countries, such as Denmark, although restrictions on their use were introduced by the Danish Veterinary and Food Administration (DVFA) in 2002 (Høg et al., 2015).

Multilocus sequence type (ST10) is an extremely diverse *E. coli* lineage found in many different host species, belonging to phylogenetic group A (Fischer et al., 2017). It was the most prevalent ST among the *E. coli* isolates submitted for whole genome sequencing (four of six isolates; 66.67%) and was overrepresented in a recent whole genome sequence analysis of porcine commensal *E. coli* isolated from two piggeries in Australia (Reid et al., 2017). ST10 is recognized as a potentially zooanthroponotic commensal clonal lineage that has also been identified as a cause of extraintestinal infections in humans in both hospital and community settings in the Netherlands and Canada. It has also been detected in poultry, wild birds, pigs and retail chicken and pork meat (Abraham et al., 2015). Previous studies have isolated an ESC-non-susceptible ST10 *E. coli* strain from a calf with diarrhea in Australia (Abraham et al., 2015), as well as dust and manure samples from piggeries in Germany (Fischer et al., 2017). Carriage by European farmers has also been previously reported (Fischer et al., 2017). However, its potential contribution to the spread of AMR between humans and animals is a very recent observation (Wang et al., 2016). Its frequent association with ESBL production, and widespread detection in humans, meat products and food animals, are important epidemiological traits (Manges and Johnson, 2012). Although the increased serum survival (*iss)* gene, recognized for its role in ExPEC infections (Miajlovic and Smith, 2014), was identified in half the porcine ST10 isolates obtained in the present study, the isolates do not conform to the molecular definition of an ExPEC strain as they do not contain ≥2 of the ExPEC associated virulence genes *papA* and/or *papC, sfa/focDE, afa/draBC, kpsM II*, and *iutA* (Guo et al., 2015).

In the absence of PMQR genes, mutational alterations in the FQ target enzymes DNA topoisomerase II and topoisomerase IV are the major mechanisms through which chromosomal resistance occurs in Gram-negative bacteria (Gruger et al., 2004). In isolates showing FQ non-susceptibility, such as the ST10 isolates identified in this study, DNA gyrase, the primary target in Gram-negative bacteria, normally possesses GyrA subunit substitutions at amino acid positions S83 and/or D87 and ParC subunit substitutions at S80 and E84 (Gruger et al., 2004). One of the most common mutations that results in high level resistance to FQs alters S83 to either L or W, which can result in an approximate 10-fold increase in MIC (Gruger et al., 2004). All three of the most common mutation sites (S83, D87 and S80) were present in the two FQ-resistant ST10 isolates (MICs > 4 μg/ml) and a single isolate above the wild-type MIC (MIC 0.5 μg/ml) obtained in this study.

The isolate belonging to the new ST7573 contained the *ipfA* virulence gene, which encodes an adhesin that plays an integral role in attachment of enteropathogenic *E. coli* (EPEC) to the gut wall and has been found to be prevalent in both clinical and commensal *E. coli* isolated from human and bovine hosts (Blum and Leitner, 2013). This gene was identified in association with the *iss* serum survival gene and *astA* toxin genes, together with genes encoding microcins and microcin immunity. In a recent study by Blum and Leitner (2013), *iss* and *astA* were the most prevalent virulence factor genes identified in *E. coli* associated with bovine mastitis (Blum and Leitner, 2013). A recent whole genome sequence comparative analysis of 103 porcine commensal *E. coli* from two piggeries in Australia identified a greater array of extraintestinal *E. coli* virulence genes in 14 ST10 isolates (Reid et al., 2017), but none would conform to the strict definition of an ExPEC strain (Guo et al., 2015). It is therefore difficult to infer if these genes are adaption genes commonly found in commensal *E. coli* or true virulence genes (Abraham et al., 2012).

Two *Salmonella* spp. isolates with MICs above the ceftiofur ECOFF were further characterized by whole genome sequencing. Interestingly, one of the isolates belonging to ST469, a sequence type commonly associated with the *Salmonella* spp. serotype Rissen. This serotype is commonly isolated from both humans and pork production systems in different parts of the world, notably Asia (Pornsukarom et al., 2015). The second ST515 (serotype Johannesburg) has been previously isolated from a mix of environmental, human and livestock isolates from Nigeria, the U.S.A and the U.K. and interestingly, from boneless camel meat in Australia (http://enterobase.warwick.ac.uk).

In conclusion, this study has identified *E. coli* isolates with MICs above the wild-type for ciprofloxacin that belong to diverse host range clonal lineages, such as ST10, in Australian piggeries, despite strict biosecurity and the absence of FQ selection pressure (Abraham et al., 2015). Such strains may have been introduced into the Australian piggery environment from an external source, possibly via humans, migratory birds or other vectors. Overall, however, the results of this study endorse the generally conservative approach to the use of CIAs in the Australian pig industry as only very low levels of non-susceptibility to these drugs were detected among both *E. coli* and *Salmonella* spp. isolates from healthy finisher pigs. This represents a baseline for benchmarking in future AMR surveillance programs.

## Author contributions

AK performed experiments, data analysis, and drafted and prepared the manuscript. JB performed experiments and analyzed data. MO performed whole genome sequencing, sequencing analysis, and was involved in manuscript preparation. TL performed whole genome sequencing. SA, DJ, PM, CM, and DT were involved in experimental design development and manuscript preparation.

### Conflict of interest statement

DT has received research funding and undertaken consultancies for Bayer, Zoetis, Merial, Virbac, Luoda Pharma, Neoculi, and IRiccorgpharm. SA has received research funding from Zoetis and Neoculi. The remaining authors declare that the research was conducted in the absence of any commercial or financial relationships that could be construed as a potential conflict of interest.
